# Effects of Dietary Crude Glycerol Supplementation on Performance, Egg Quality, and Yolk Fatty Acids in Laying Hens: A Systematic Review and Meta-Analysis

**DOI:** 10.3390/ani16101486

**Published:** 2026-05-12

**Authors:** Panneepa Sivapirunthep, Rasheed Olayiwola Sulaimon, Katatikarn Sahatsanon, Anuraga Jayanegara, Pattraphorn Patthararangsarith, Chanporn Chaosap

**Affiliations:** 1 Department of Agricultural Education, School of Industrial Education and Technology, King Mongkut’s Institute of Technology Ladkrabang, Bangkok 10520, Thailand; panneepa.si@kmitl.ac.th (P.S.); pattraphorn.pa@kmitl.ac.th (P.P.); 2 Doctoral Program in Innovative Tropical Agriculture, Department of Agricultural Education, School of Industrial Education and Technology, King Mongkut’s Institute of Technology Ladkrabang, Bangkok 10520, Thailand; 67036072@kmitl.ac.th (R.O.S.); 65036095@kmitl.ac.th (K.S.); 3 Department of Animal Science, Faculty of Agriculture, University of Abuja, Abuja 902101, Nigeria; 4 Department of Animal Nutrition and Feed Technology, Faculty of Animal Science, IPB University, Bogor 16680, Indonesia; anuragaja@apps.ipb.ac.id

**Keywords:** egg mass, energy substitution, layer productivity, heterogeneity, meta-analysis

## Abstract

This study evaluated the effects of dietary crude glycerol supplementation on laying performance, egg quality traits, and egg yolk fatty acid composition in laying hens. A systematic search of peer-reviewed literature from major scientific databases identified thirteen eligible studies. Effect sizes were calculated as mean differences using random-effects models, and subgroup and meta-regression analyses were conducted to assess the influence of inclusion level, hen age, strain, and dose–response relationships. Moderate inclusion of crude glycerol (≤4%) significantly increased egg production (MD = +0.71 percentage points; *p* = 0.019) and egg mass (MD = +1.08 g; *p* = 0.007). External egg quality traits, including shell thickness, shell breaking strength, and shell percentage, were not significantly affected, while albumen height decreased slightly (MD = −0.14 mm; *p* = 0.004) and Haugh unit remained unchanged. Crude glycerol supplementation also increased linoleic acid, total unsaturated fatty acids, and total polyunsaturated fatty acids (*p* < 0.001). Responses varied with inclusion level, hen age, and strain, and a positive dose–response relationship between crude glycerol level and yolk linoleic acid was observed in white-egg strains. Overall, moderate crude glycerol inclusion can partially replace conventional dietary energy sources with minimal adverse effects on egg quality.

## Simple Summary

The rising cost of poultry feed has increased interest in alternative energy sources that can maintain productivity without compromising egg quality. Crude glycerol, a by-product of biodiesel production, has been proposed as a potential feed ingredient for laying hens. However, results from individual studies have been inconsistent. This study combined data from several published experiments to better understand the overall effects of crude glycerol in layer diets. The findings showed that including moderate levels of crude glycerol in the diet can slightly improve egg production and egg mass without negatively affecting most egg quality traits. Some minor changes in internal egg quality were observed, but they were not considered detrimental. In addition, crude glycerol increased the levels of certain beneficial fatty acids in egg yolk, which may have nutritional value for consumers. Overall, crude glycerol can be used as a partial replacement for conventional energy sources in laying hen diets. However, the effects may vary depending on the quality of the glycerol, the diet composition, and the age or type of birds. These results provide useful information for improving feed efficiency and sustainability in egg production systems.

## 1. Introduction

The poultry industry is under increasing pressure to reduce feed costs while maintaining high levels of production efficiency and product quality. Because energy ingredients account for a substantial proportion of diet formulation costs in laying hen systems, there is growing interest in alternative feed resources that can partially replace conventional energy sources without impairing performance. In laying hens, dietary energy plays a central role in regulating nutrient partitioning, egg production, and egg quality attributes, making the identification of alternative energy sources a priority for both nutritionists and producers [1,2]. Variations in dietary energy density can influence feed intake regulation, egg mass output, and internal egg quality traits.

Among potential alternatives, agro-industrial by-products with high energetic value have received increasing attention in layer nutrition. Crude glycerol (CG), a co-product generated during biodiesel production, has emerged as a promising candidate due to its high glycerol content, glucogenic potential, and expanding availability from the biodiesel industry [3,4,5]. CG primarily contains glycerol along with variable amounts of water, mineral salts, free fatty acids, and residual methanol, depending on processing conditions [6]. Glycerol is rapidly absorbed in the gastrointestinal tract and enters glycolytic and gluconeogenic pathways, thereby serving as a readily utilized energy substrate for poultry [6,7].

In laying hens, glycerol metabolism is closely associated with hepatic function, as the liver is the principal site of lipid synthesis and yolk precursor formation [6,8]. Adequate hepatic energy supply supports the synthesis of very-low-density lipoproteins and vitellogenin, which are subsequently transported to the ovary for yolk deposition and follicular development [9,10]. Several experimental studies have evaluated the inclusion of CG in layer diets, primarily focusing on production performance indicators such as egg production, feed intake, feed conversion ratio, and egg mass. Some reports suggest that moderate CG inclusion (generally ≤4–6%) can maintain or improve laying performance without detrimental effects [11,12]. In contrast, higher inclusion levels have occasionally been associated with reduced feed intake, altered shell quality, or changes in certain egg traits, potentially related to palatability, electrolyte balance, or variability in CG purity [13,14]. These inconsistent findings indicate that responses to CG may depend on factors such as glycerol purity, dietary inclusion rate, hen age, basal diet composition, and experimental duration.

Beyond production performance, egg quality traits, including egg weight, albumen height, Haugh unit, yolk colour, and shell characteristics, are critical determinants of consumer acceptance and market value [15,16]. Dietary energy sources can influence osmotic balance, protein deposition, and lipid metabolism during egg formation; however, individual studies evaluating CG have reported conflicting outcomes, particularly for albumen-related traits [17,18,19]. In addition, increasing attention has been directed toward egg yolk fatty acid composition due to its relevance to human nutrition and functional egg production. Yolk fatty acid profiles are strongly influenced by dietary lipid sources and hepatic metabolism in laying hens [20]. Although CG does not directly supply fatty acids, its role in hepatic energy metabolism may indirectly affect yolk lipid deposition. Nevertheless, available studies examining saturated, monounsaturated, and polyunsaturated fatty acids, including key components such as linoleic acid and docosahexaenoic acid (DHA), remain limited and report variable responses [12,18,21].

The composition of CG has been reported to include glycerol, methanol, moisture, ash, crude protein, and crude fat [6]. CG supplementation has been reported to exert minimal to moderate effects on the basic chemical composition of eggs, including dry matter, crude protein, lipid, and mineral content, with responses largely dependent on inclusion level and diet formulation [3]. Most studies indicate that moderate inclusion levels do not substantially alter proximate composition, suggesting that CG can be incorporated as an alternative energy source without markedly affecting the nutritional composition of eggs [13]. Egg sensory attributes, including yolk colour, taste, and odour, are important determinants of consumer acceptance and market preference. Consumers often use these sensory characteristics, particularly yolk colour, as indicators of egg quality and freshness [22]. Yolk colour is largely influenced by the type and level of carotenoid pigments present in the hen’s diet, which are absorbed and deposited in the yolk [23]. Consequently, dietary composition can indirectly affect sensory qualities through changes in nutrient utilization and pigment deposition, thereby influencing consumer perception and acceptance of eggs [24]. Furthermore, technological characteristics such as albumen pH and related quality indices can be influenced by metabolic and nutritional factors that regulate protein structure and water-holding capacity. Albumen quality is largely dependent on its protein composition, particularly the integrity of protein networks such as ovomucin, which determines gel structure, viscosity, and functional properties, including water-holding capacity. These properties are influenced by dietary factors, bird physiology, and environmental conditions, which, in turn, affect albumen pH and overall egg quality [25,26]. Beyond egg characteristics, CG may modulate physiological responses in laying hens, as reflected in blood biochemical parameters [5]. Previous studies have shown that glycerol supplementation can influence indicators of liver function and energy metabolism, including enzymes and circulating metabolites associated with gluconeogenesis and lipid utilization [27]. However, these responses are inconsistent and appear to depend on inclusion level, glycerol purity, and the physiological stage of the birds.

Although numerous studies have evaluated the use of crude glycerol in laying hens, the overall effects of CG supplementation remain unclear. This is not only due to heterogeneity in experimental conditions but also reflects several key limitations in the existing literature, including the lack of systematic dose–response evaluation, insufficient control and reporting of crude glycerol composition (e.g., glycerol purity, residual methanol, and mineral content), and the limited integration of mechanistic insights related to energy metabolism and hepatic lipid partitioning. These gaps limit the ability to draw consistent and biologically meaningful conclusions from individual studies. Therefore, a quantitative synthesis that integrates these sources of variability is necessary to provide a more robust and comprehensive evaluation of CG supplementation in laying hens.

Meta-analysis provides an appropriate quantitative framework to address these inconsistencies; however, comprehensive syntheses focused specifically on crude glycerol use in laying hens remain scarce. Existing studies are largely limited to individual experimental reports, often with small sample sizes and varying designs, and few attempts have been made to integrate outcomes across production performance, egg quality traits, and yolk fatty acid composition within a single analytical framework. In addition, previous work has rarely incorporated systematic evaluation of moderating factors such as dietary inclusion level, hen age, and genetic strain, nor has it consistently explored potential dose–response relationships. Therefore, the present study aimed to address these limitations by synthesizing available evidence using a meta-analytical approach that integrates multiple response variables and incorporates subgroup and meta-regression analyses to better explain sources of variability and provide a more comprehensive evaluation of crude glycerol supplementation in laying hens. Accordingly, this study quantified the effects of dietary crude glycerol supplementation on production performance, egg quality traits, and egg yolk fatty acid composition in laying hens, while evaluating the influence of key moderating factors, including inclusion level, laying age, and strain. We hypothesized that moderate CG inclusion would increase laying performance by providing glucogenic substrates to support energy metabolism, thereby enhancing laying performance, while exerting no significant effects on eggshell quality.

## 2. Materials and Methods

### 2.1. Literature Search and Data Sources

A systematic literature search was conducted between December 2025 and February 2026 to identify peer-reviewed studies evaluating the effects of dietary CG supplementation in laying hens, including all relevant publications available up to February 2026. The search process followed the Preferred Reporting Items for Systematic Reviews and Meta-Analyses (PRISMA) guidelines. The databases Web of Science, Scopus, and PubMed were systematically searched for relevant articles using the search strategy employed combinations of keywords using Boolean operators (AND/OR), as follows (“crude glycerol” OR “glycerol” OR “glycerin”) AND (“laying hen” OR “layer”) AND (“egg production” OR “egg quality” OR “yolk fatty acid*”). No publication year restrictions were applied, as this represents the first meta-analysis synthesizing evidence on CG supplementation in laying hens. Google Scholar was also used as a supplementary source to identify potentially relevant studies, and the results were sorted by relevance and screened to ensure consistency and manageability. Only studies published in English in peer-reviewed journals were considered. Grey literature, including conference proceedings, theses, and unpublished data, was excluded to ensure the reliability and consistency of the dataset. In addition, the reference lists of all retrieved articles were manually screened to identify any further eligible studies not captured in the initial database search.

### 2.2. Eligibility Criteria

Studies were included when they satisfied the following requirements: (1) peer-reviewed original articles written in English; (2) experiments performed in laying hens; (3) dietary treatments involving CG compared with a control diet without CG; and (4) reporting at least one relevant outcome such as production performance, egg quality parameters, or yolk fatty acid composition with statistical information to permit effect size calculation, including mean values, sample sizes, and a measure of variability (standard deviation or standard error).

**Figure 1:**
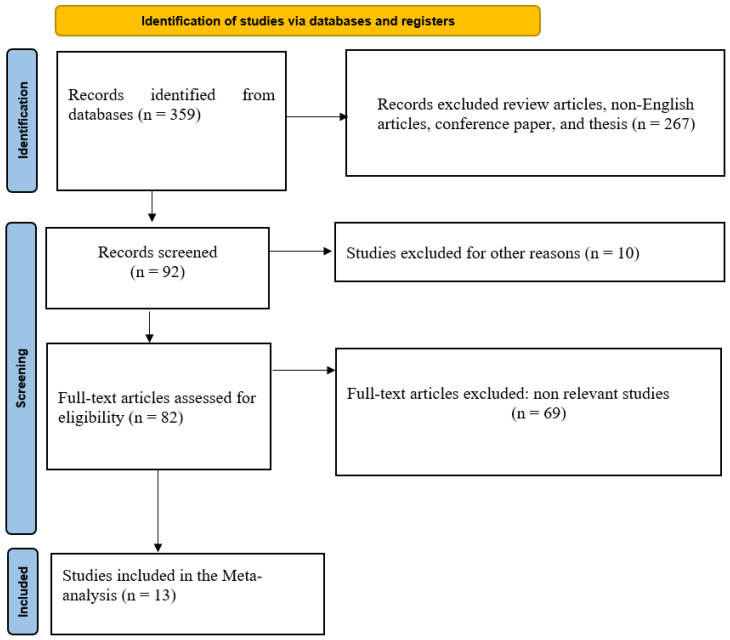
Literature search and selection process following PRISMA 2020 guidelines.

Studies were excluded if they: (1) involved broilers or other non-laying hen poultry species; (2) evaluated purified glycerol without clear identification as CG; (3) lacked a suitable control treatment; (4) did not provide extractable numerical data; (5) were reviews, conference proceedings, theses, or other non-peer-reviewed materials; or (6) represented duplicate reports of the same experimental dataset. When multiple publications described the same trial, the most comprehensive dataset was used for analysis. The study selection process is illustrated in the PRISMA flowchart (Figure 1). The PRISMA checklist was used to ensure the inclusion of all relevant information in the analysis (Supplementary Materials Data S1).

### 2.3. Risk of Bias Assessment

The methodological quality of the included studies was assessed using criteria adapted from the SYRCLE risk of bias tool for animal studies. The following domains were evaluated: sequence generation (randomization), allocation concealment, blinding of personnel, blinding of outcome assessment, incomplete outcome data, and selective outcome reporting. Each item was judged as low, high, or unclear risk of bias based on information reported in the original publications. Where methodological details were insufficient, the risk was classified as unclear (Supplementary Materials Data S2).

### 2.4. Data Extraction

Information from each eligible study was systematically collected using a predefined extraction template. When available, the following study characteristics were recorded: first author, publication year, country of origin, laying hen strain, bird age, experimental duration, CG inclusion level, key diet characteristics, and the number of experimental units per treatment. Relevant outcome data were also extracted for subsequent quantitative synthesis.

For each outcome, mean values, corresponding measures of dispersion (standard deviation, standard error, or pooled error), and sample sizes were compiled to enable effect size calculation. Reported standard errors were converted to standard deviations using conventional formulas. In experiments with multiple CG inclusion levels, each treatment was compared independently with its corresponding control group. Although this approach is commonly applied in meta-analyses, it may introduce some degree of non-independence among effect sizes. Given the limited number of studies and the variability in reporting formats, more advanced approaches, such as pooled treatment groups or multilevel modelling, were not feasible in this study. All extracted datasets were organized and coded in Microsoft Excel prior to statistical synthesis.

### 2.5. Outcome Variables

The extracted outcomes were classified into three predefined categories. Production performance variables comprised egg production (EP, %), feed intake (FI, g/hen/day), and feed conversion ratio (FCR, kg feed/kg egg). Egg quality traits included egg weight (EW, g), egg mass (EM, g), albumen height (AH, mm), Haugh unit (HU), yolk colour score (YC), eggshell thickness (EST, mm), eggshell breaking strength (ESBS, N), and eggshell percentage (ES, %). Egg yolk fatty acid outcomes consisted of major individual fatty acids and grouped lipid indices (e.g., PUFA), expressed as a percentage of total fatty acids.

### 2.6. Statistical Analysis

All meta-analyses were conducted using OpenMEE software (v1.0.0). Treatment effects of CG supplementation were expressed as mean differences (MD) between the CG and control groups because most outcomes were reported on comparable measurement scales. Results are presented with 95% confidence intervals (CI), and statistical significance was declared at *p* < 0.05. A random-effects model was applied using the DerSimonian–Laird method [28] to account for anticipated variability among studies. Between-study heterogeneity was assessed using Cochran’s Q statistic and quantified with the I^2^ index; values of approximately 25%, 50%, and 75% were interpreted as low, moderate, and high heterogeneity, respectively.

To explore potential sources of heterogeneity, prespecified subgroup analyses were conducted based on (1) dietary CG inclusion level (≤4% vs. >4%), (2) laying age (≤57 weeks vs. >57 weeks), and (3) laying hen strain (brown-egg vs. white-egg). Subgroup analyses were performed only for outcomes with sufficient numbers of comparisons and clear biological relevance to avoid overinterpretation from sparse data. Accordingly, subgroup evaluation was limited to EP and FCR for performance, EM and HU for egg quality, and C18:2 and total polyunsaturated fatty acids (PUFA) content for yolk fatty acid composition. In addition, meta-regression analysis was conducted to assess the relationship between CG inclusion level and C18:2.

Potential publication bias was assessed through visual inspection of funnel plots combined with Egger’s regression test. Although funnel plots and Egger’s test are widely used to detect publication bias, additional methods such as trim-and-fill were not applied due to the limited number of studies available for several outcomes, which may reduce the reliability of these approaches. The assessment was conducted for outcomes included in the subgroup analyses to ensure adequate statistical power. Egger’s test was performed using MetaWin 3 software [29], with statistical significance set at *p* < 0.05 [30]. A significant Egger’s test indicates potential small-study effects or funnel plot asymmetry, which may suggest publication bias. When evidence of bias was detected, sensitivity analyses were conducted sequentially, by excluding individual studies to evaluate their influence on the pooled effect size and to determine the robustness of the overall findings under conditions of high heterogeneity [31]. The relatively small number of included studies may reduce statistical power and limit the robustness of some findings, particularly in subgroup analyses and publication bias assessments.

## 3. Results

### 3.1. Overview of Included Studies

The main characteristics of the studies included in the meta-analysis are summarized in Table 1. A total of 13 peer-reviewed experiments were included, published between 2009 and 2023. The studies were conducted across several countries, including Poland, Turkey, Hungary, the Czech Republic, Brazil, and Spain, reflecting a wide range of production conditions. The experiments included several commercial laying hen strains, predominantly brown-egg genotypes (e.g., Bovans Brown, Lohmann Brown, Hy-Line Brown, Hisex Brown, and Tetra SL), with fewer white-egg strains (Hy-Line W36). The studied laying age of hens ranged from approximately 20 to 90 weeks, encompassing early, peak, and late laying phases. Most studies evaluated birds during the early-to-mid laying period (≤57 weeks), whereas only two studies represented late-cycle hens. Dietary CG inclusion levels varied widely across studies, ranging from 1% to 14% of the diet. Study duration varied widely, from short metabolism trials to long-term production experiments lasting more than 100 days or spanning multiple laying cycles. The number of birds per study ranged widely, reflecting differences in experimental design and scale. Across the dataset, the most frequently reported outcomes were production performance (including EP, FI, and FCR) and egg quality traits, with some additional studies reporting egg yolk fatty acid profiles. The chemical composition of CG, including glycerol purity and residual methanol content, was variably reported among studies.

### 3.2. Production Performance

Dietary crude glycerol supplementation significantly influenced selected production performance parameters in laying hens (Table 2). EP was modestly but higher in hens receiving CG, with a mean difference of 0.71 percentage points (95% CI: 0.117 to 1.296; *p* = 0.019). CG supplementation did not affect FI (MD = 0.48 g/hen/day; 95% CI: −0.568 to 1.517; *p* = 0.372), suggesting that voluntary feed consumption remained stable. FCR was not significantly reduced in CG-fed hens (MD = −0.002; 95% CI: −0.020 to 0.015; *p* = 0.813), indicating unimproved feed efficiency. High heterogeneity was observed for all production traits (I^2^ = 88–96%), suggesting substantial variability across studies.

### 3.3. Egg Quality Traits

The pooled effects of CG supplementation on egg quality traits are presented in Table 3. Dietary CG supplementation increased EM in laying hens (MD = +1.08 g; 95% CI: 0.303 to 1.862; *p* = 0.007). AH was reduced (MD = −0.137 mm; 95% CI: −0.231 to −0.042; *p* = 0.004), whereas HU showed a numerical decline that did not reach statistical significance (MD = −0.408; 95% CI: −0.879 to 0.063; *p* = 0.089). CG supplementation did not significantly affect EW, YC, EST, ESBS, or ES (*p* > 0.05). Heterogeneity ranged from low to very high across egg quality traits (I^2^ = 0–93%), with EW and HU showing particularly high variability among studies.

### 3.4. Egg Yolk Fatty Acid Composition

Supplementation with dietary CG significantly affected several egg yolk fatty acids and lipid fractions (Table 4). Linoleic acid (C18:2) was increased by +1.77% (*p* < 0.001), accompanied by an increase in total polyunsaturated fatty acids (PUFA; +0.80%) and total unsaturated fatty acids (UFA; +1.67%) (*p* < 0.001). Conversely, palmitic acid (C16:0), stearic acid (C18:0), and total saturated fatty acids (SFA) were significantly reduced (*p* < 0.05). Minor but increases were observed for myristic (C14:0), oleic (C18:1), behenic (C22:0), and lignoceric acids (C24:0). High heterogeneity was detected for most fatty acid outcomes, with I^2^ exceeding 95% in most cases, indicating substantial variability among studies.

### 3.5. Subgroup Analysis and Meta-Regression

Subgroup analyses suggested possible variation in response to CG by inclusion level, laying age, and strain (Table 5). FCR, EP, HU, and PUFA were not significantly affected by the inclusion level of CG. EM increased significantly at CG inclusion ≤ 4% (MD = +1.27 g; *p* = 0.014), whereas only a numerical increase was observed at higher inclusion levels (MD = +1.01 g; *p* = 0.059), which did not reach statistical significance. Similarly, linoleic acid (C18:2) increased at CG inclusion ≤ 4% (MD = +0.60%; *p* = 0.034) but was not significantly affected at higher inclusion levels. Age-based subgroup analysis indicated that EP was higher in hens older than 57 weeks (MD = +4.63%; *p* < 0.001), whereas there was no significant response in younger hens. Similarly, EM was significantly improved in hens >57 weeks (MD = +2.68 g; *p* < 0.001), whereas no effect was detected in younger birds. According to strain-based subgroup analysis, white-egg strains showed a significant reduction in FCR (MD = –0.022; *p* = 0.006), indicating improved feed efficiency. Brown-egg strains showed an increase in EP (+1.69%; *p* < 0.001) and a decrease in HU (–1.005%; *p* < 0.001). Both brown- and white-egg strains demonstrated statistically increases in EM (+0.95 and +1.27 g, respectively; *p* < 0.05), linoleic acid (C18:2) (MD = +0.60 and +1.22, respectively; *p* < 0.05 and *p* < 0.001), and PUFA (MD = +0.74 and +0.79, respectively; *p* < 0.05 and 0.001).

**Figure 2:**
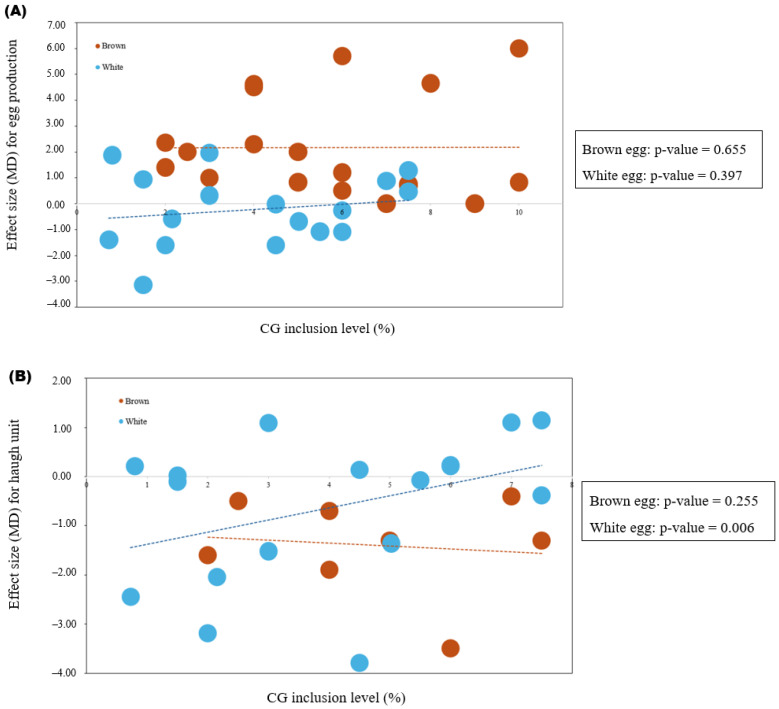

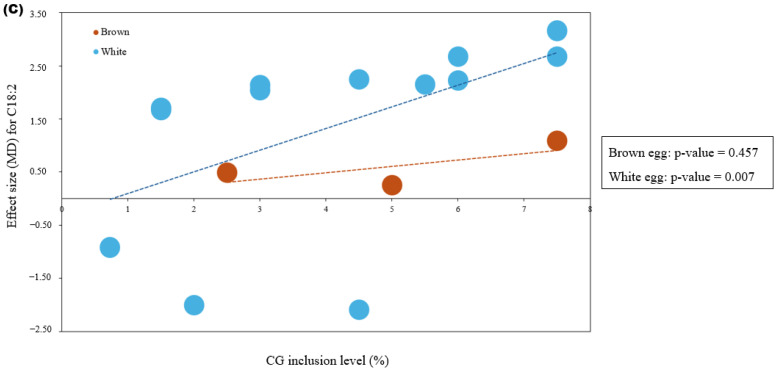
Meta-regression analyses illustrating the relationship between crude glycerol (CG) inclusion level and (**A**) egg production, (**B**) Haugh unit, and (**C**) yolk linoleic acid (C18:2) in brown- and white-egg laying hen strains. Each point represents an individual comparison, with solid and dashed lines indicating fitted regression lines for brown- and white-egg strains, respectively. Corresponding *p*-values for each strain are presented within each panel.

Meta-regression analysis revealed no significant association between CG inclusion level, EP, HU, and yolk C18:2 content in brown-egg strains, whereas a significant positive relationship was observed in white-egg strains for HU and C18:2 (*p* < 0.01) (Figure 2).

### 3.6. Publication Bias

**Figure 3:**
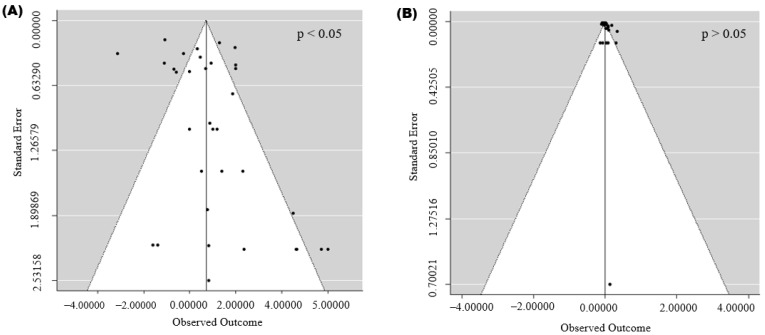

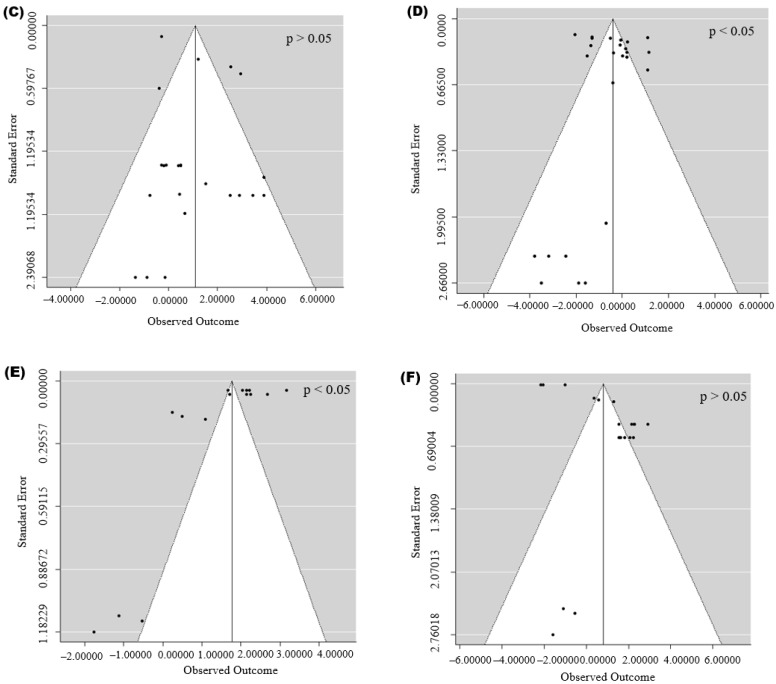
Funnel plots illustrating the effects of dietary crude glycerol on (**A**) egg production, (**B**) feed conversion ratio, (**C**) egg mass, (**D**) Haugh unit, (**E**) C18:2, and (**F**) PUFA.

Publication bias was evaluated for EP, FCR, EM, HU, C18:2, and PUFA using funnel plots and Egger’s regression test (Figure 3). Visual inspection indicated generally symmetrical distributions around the pooled effect sizes for FCR, EM, and PUFA outcomes, with effect estimates evenly scattered on both sides of the central line, suggesting minimal small-study effects. Egger’s test confirmed the absence of significant publication bias for FCR, EM, and PUFA (*p* > 0.05). For EP, HU, and C18:2, although Egger’s test was significant, the leave-one-out sensitivity analysis showed that sequentially removing individual studies did not materially alter the pooled estimate, indicating that the overall findings were robust.

## 4. Discussion

### 4.1. Production Performance

This meta-analysis showed that dietary CG slightly increased EP, while FI and FCR remained unchanged. Although the increase in EP (+0.71 percentage points) was statistically significant, the magnitude of this effect is relatively small; however, in commercial layer operations with thousands of birds, even marginal increases in laying rate can translate into a substantial cumulative number of eggs, suggesting potential practical relevance at the flock or industry scale. Moreover, given that EP is a tightly regulated biological process, such a small but consistent difference may reflect a sensitive and reproducible physiological response to the treatment. This modest response likely reflects the role of glycerol as a readily utilizable glucogenic substrate that supports the energy demands of egg formation without stimulating additional feed consumption. The unchanged FI further indicates that CG inclusion at the tested levels does not impair diet palatability. The relatively small change in EP may be attributed to glycerol functioning as a readily utilizable glucogenic energy source that contributes to dietary energy supply. As reported by Boso et al. [12], glycerol is rapidly absorbed in the intestine via both active and passive transport mechanisms and can provide readily available energy. Consequently, inclusion of CG in laying hen diets appears sufficient to maintain energy supply without adversely affecting production performance; however, its capacity to meaningfully enhance productivity may be limited.

An increase in EP did not translate into improved FCR, indicating a limited effect on overall feed efficiency. Differences in dietary energy balance among studies may be partially partitioned toward maintenance functions rather than egg output, as energy allocation in laying hens is known to depend on physiological state and nutrient availability [3]. Energy utilization in laying hens involves the partitioning of metabolizable energy into maintenance, growth, and egg production. Maintenance requirements, largely associated with basal metabolism and heat production, are prioritized before energy is allocated to productive functions such as egg formation [38]. Studies have shown that metabolizable energy intake is distributed among these components, with distinct energetic costs associated with egg mass production and body weight gain [39]. Experimental approaches have further demonstrated that maintenance and egg production energy requirements can be quantified separately, highlighting their physiological independence [40]. Moreover, the proportion of energy allocated to these functions varies with age, reproductive status, and feeding systems, indicating that energy partitioning is dynamic rather than fixed [41]. Accurate understanding of these relationships is essential for optimizing feeding strategies and improving production efficiency in laying hens. Consistent with the present findings, Yalçın et al. [17], Boso et al. [12], Kanbur et al. [19], and Fontinele et al. [36] reported no statistical differences in FI or FCR between control hens and those fed CG-supplemented diets. In contrast, Németh et al. [33] observed an increase in FCR when glycerol was included at 10%, suggesting that excessive inclusion levels may negatively affect efficiency. The high heterogeneity observed (I^2^ = 88–96%) indicates considerable variability among studies, likely due to differences in experimental conditions. Subgroup analyses showed that CG inclusion level contributed to inconsistent responses, with lower levels generally supporting better performance than higher levels. Hen age also influenced the results, as younger hens appeared to respond more consistently than older hens, likely due to differences in metabolic efficiency. Additionally, strain differences may explain part of the heterogeneity, reflecting variation in nutrient utilization and production characteristics. These findings suggest that the observed heterogeneity is mainly driven by biological and nutritional factors rather than random variation. Therefore, although CG appears safe as a partial energy substitute in laying hen diets, its productive benefits are likely context-dependent. In addition, the observed improvements in production performance are modest and should be interpreted cautiously regarding their practical significance.

### 4.2. Egg Quality Traits

The pooled analysis showed that dietary CG increased EM, whereas most egg quality traits were unaffected. Although EM improved significantly (+1.08 g), the magnitude of this effect remains relatively small and may have limited practical importance in commercial egg production systems. This response may reflect the contribution of glycerol, a readily available glucogenic energy source, to egg formation. Because EM is closely associated with dietary energy supply, the rapid utilization of glycerol may have enhanced egg output without altering FI.

AH was slightly reduced, and HU showed a non-significant downward trend. The observed reduction in AH, although small, was statistically significant and suggests subtle changes in internal egg quality. Although the extent of this change is small, it should not be disregarded, as albumen quality is an important parameter influencing egg freshness and consumer acceptance. Possible explanation involves the role of glycerol in osmotic regulation and water balance [3]. Glycerol is a highly osmotic molecule that can influence water distribution and may alter the hydration state of albumen proteins during egg formation [6]. Changes in osmotic pressure could affect the viscosity and structural integrity of thick albumen, thereby contributing to reduced albumen height [42]. In addition, glycerol serves as a glucogenic substrate that supports hepatic energy metabolism, which is closely linked to protein synthesis and nutrient partitioning in laying hens [43]. Albumen proteins are synthesized primarily in the magnum, and their deposition depends on both amino acid availability and systemic metabolic status [44]. Alterations in energy supply and nutrient partitioning may therefore influence the rate or efficiency of albumen protein deposition. Furthermore, water transport into the albumen is regulated by osmotic gradients and protein matrix formation, and any shift in these processes may affect albumen consistency [45,46]. However, the exact mechanisms linking glycerol metabolism to albumen quality remain unclear, and the observed changes were relatively small. Previous studies have reported inconsistent responses of albumen traits to glycerol inclusion [17,19]. For example, Erol et al. [47] observed reduced AH only at the highest glycerol inclusion level (100 g/kg), suggesting a dose-dependent response. The authors noted that higher glycerol inclusion required reformulation of other dietary components (e.g., corn, soybean oil, and soybean products), which may have indirectly influenced albumen quality.

EW, YC, and eggshell quality parameters (EST, ESBS, and ES) were not significantly affected, indicating that CG can partially replace dietary energy sources without compromising shell formation or yolk pigmentation. These results suggest that calcium metabolism and shell matrix deposition remain largely stable with moderate levels of CG inclusion in nutritionally balanced diets. Heterogeneity ranged from low to very high (I^2^ = 0–93%), indicating that responses may vary with CG composition, inclusion level, hen age and strain, and basal diet formulation. Cumulatively, CG appears suitable as a partial energy substitute in laying hen diets, with generally limited effects on egg quality; however, the observed reduction in albumen height suggests that certain internal quality parameters may be sensitive to dietary inclusion levels.

### 4.3. Egg Yolk Fatty Acids

Dietary CG altered the yolk lipid profile primarily by increasing the proportion of unsaturated fatty acids. Linoleic acid (C18:2), total PUFA, and total UFA were significantly elevated, suggesting a tendency toward increased deposition of unsaturated fatty acids into the yolk. As glycerol serves both as a glucogenic substrate and as the glycerol backbone for triglyceride synthesis, its availability may facilitate hepatic lipid transport and subsequent incorporation of dietary fatty acids into the egg [3,21]. Concurrently, palmitic acid (C16:0), stearic acid, and total SFA declined, reflecting a shift away from saturated fatty acids, a pattern that may be considered nutritionally favourable. Minor but significantly higher values were also detected for myristic (C14:0), oleic (C18:1), behenic (C22:0), and lignoceric acids (C24:0), although the biological relevance of these changes is likely limited. The observed changes are likely mediated through CG role in hepatic energy metabolism and lipid synthesis pathways [20]. Following absorption, glycerol is converted to glycerol-3-phosphate and enters gluconeogenesis, contributing to glucose production and overall energy availability in the liver. This enhanced glucogenic flux can influence hepatic lipid metabolism, as the liver is the central organ responsible for de novo lipogenesis and lipid transport in laying hens [48,49]. In avian species, lipids destined for yolk deposition are synthesized or re-esterified in the liver and exported as very-low-density lipoproteins (VLDL) and vitellogenin, which are subsequently transported via the bloodstream to the developing ovarian follicles [9,50]. Because glycerol does not directly supply fatty acids, the observed changes in yolk lipid composition are likely mediated indirectly through alterations in hepatic energy metabolism. As a glucogenic substrate, glycerol may increase energy availability, potentially reducing reliance on β-oxidation of dietary unsaturated fatty acids and thereby favouring their deposition in yolk lipids [6,20,48]. However, yolk fatty acid composition is strongly influenced by the type and composition of dietary lipids in the basal diets. Variations in fat sources, such as vegetable oils or animal fats, can substantially affect yolk lipid profiles. Therefore, the responses observed in the present analysis likely reflect interactions between glycerol-derived energy and the underlying dietary lipid composition, rather than a direct effect of crude glycerol itself. In addition, inconsistent reporting of dietary fatty acid profiles across studies limited our ability to quantify this factor.

Most fatty acid outcomes exhibited very high heterogeneity (I^2^ > 95%), indicating substantial between-study variation. Differences in CG purity and fatty acid composition, basal dietary fat sources, inclusion level, hen genotype and age, and analytical methodology likely contributed to this variability. In total, CG may contribute to modest changes in yolk fatty acid composition; however, the high heterogeneity indicates that these responses are highly dependent on dietary context and should be interpreted cautiously.

### 4.4. Subgroup Analysis and Meta-Regression

The subgroup evaluation suggests that the response to CG is influenced by inclusion level, hen age, and genetic strain. The increase in egg mass at ≤4% CG indicates that moderate supplementation may optimize the utilization of dietary energy, whereas higher inclusion levels provided no additional benefit. This plateau pattern is consistent with the possibility that glycerol mainly acts as an alternative energy source; once energy requirements are satisfied, further inclusion yields limited productive gains. A comparable trend was observed for linoleic acid (C18:2), which increased only at the lower CG level, possibly reflecting interactions between glycerol-derived energy and lipid metabolism. Age-related differences were observed, although these findings should be interpreted with caution due to variability among studies. Hens older than 57 weeks showed marked improvements in EP and EM, whereas younger birds did not respond. Older hens may benefit more from supplemental glucogenic energy due to age-associated declines in metabolic efficiency or nutrient utilization, while younger hens at peak production likely already meet their energy needs [51,52]. Genotype also modified the response. White-egg strains exhibited improved FCR, whereas brown-egg strains mainly showed higher EP accompanied by a modest reduction in HU. These contrasting patterns likely reflect inherent genetic differences in nutrient partitioning and egg formation characteristics between strain types. Nevertheless, both strain groups demonstrated consistent increases in EM and yolk unsaturated fatty acids, suggesting that the metabolic effects of CG may be broadly similar across genotypes, although further evidence is needed.

For yolk linoleic acid (C18:2) and Haugh unit, Meta-regression indicated that the relationship between CG inclusion and response variables differed by strain. In both cases, no significant association was observed in brown-egg hens, whereas a significant relationship was detected in white-egg hens. Specifically, C18:2 showed a positive association with increasing CG inclusion, while the Haugh unit exhibited a negative association in white-egg strains. These findings suggest a greater sensitivity of white-egg genotypes to dietary CG, potentially reflecting differences in hepatic lipid metabolism, nutrient partitioning, and albumen protein dynamics. The increase in C18:2 may be linked to enhanced incorporation of dietary unsaturated fatty acids into yolk lipids under increased glucogenic energy supply, while the reduction in Haugh unit could indicate subtle alterations in albumen structure, possibly related to osmotic balance or protein deposition processes influenced by energy metabolism [42,53,54]. In contrast, egg production did not show a significant linear relationship with CG inclusion level in either strain, indicating that the response of production performance to CG is not strongly dose-dependent within the evaluated range. This lack of association suggests that once dietary energy requirements are met, additional glycerol inclusion may not further stimulate egg output [12]. Furthermore, the absence of a clear dose–response relationship may reflect the influence of other confounding factors, such as diet formulation, glycerol purity, and bird physiological status, which could mask incremental effects of increasing CG levels.

Despite the statistically significant findings observed in the present meta-analysis, it is important to distinguish between statistical significance and biological relevance. The relatively small magnitude of changes in key performance traits, such as egg production and egg mass, suggests that the practical benefits of CG supplementation under commercial conditions may be limited. Furthermore, the high heterogeneity observed across studies highlights the influence of unaccounted factors and reinforces the need for cautious interpretation of the pooled estimates.

### 4.5. Publication Bias

Evaluation of publication bias generally supported the stability of the pooled estimates. Funnel plots for FCR, EM, and PUFA were largely symmetrical, and Egger’s tests were non-significant, indicating little evidence of small-study effects. Although Egger’s test suggested potential asymmetry for EP, HU, and C18:2.

### 4.6. Study Limitations

Several limitations should be considered when interpreting the present findings. First, substantial between-study heterogeneity was observed for several outcomes (I^2^ often > 90%), indicating considerable variability in responses. Although subgroup and meta-regression analyses partially explained this variation (e.g., inclusion level, age, and strain), other important sources, such as crude glycerol composition (including purity and residual methanol), basal diet formulation, and experimental duration, could not be quantitatively evaluated due to inconsistent reporting across studies. Second, some subgroup and meta-regression analyses were based on a limited number of studies, which may have reduced statistical power and the precision of moderator estimates. Similarly, the relatively small overall number of included studies may affect the robustness of certain outcomes, particularly those with high heterogeneity. Third, potential dependency arising from multiple comparisons within individual studies (shared control groups) may have influenced variance estimation, although this approach is commonly applied in meta-analytical research. Finally, most included experiments were conducted under controlled research conditions, and variation in management practices, diet formulation, and production environments may limit the direct applicability of these findings to commercial systems. Therefore, the pooled estimates should be interpreted cautiously, and further well-controlled studies with standardized reporting are needed to strengthen the evidence base.

## 5. Conclusions

Crude glycerol can be incorporated into laying hen diets as an alternative energy source, with generally neutral to modest effects on production performance. This meta-analysis identified small but statistically significant increases in egg production and egg mass, while most external egg quality traits were unaffected. Although albumen height showed some sensitivity, particularly at higher inclusion levels, overall internal quality, as reflected by Haugh unit, remained largely stable. Crude glycerol supplementation was also associated with shifts in yolk lipid composition, including increases in linoleic acid and total polyunsaturated fatty acids; however, these responses are likely influenced by the composition of the basal diet and should not be attributed solely to glycerol. Subgroup analyses indicated that responses vary with inclusion level, hen age, and genetic strain, highlighting the importance of physiological stage and dietary context. Overall, moderate inclusion levels tended to produce more consistent responses across studies; however, this should not be interpreted as a definitive recommendation. The effects of crude glycerol are dependent on its quality, dietary formulation, and production conditions. Therefore, further standardized experiments and economic evaluations under commercial settings are required before establishing firm guidelines for its inclusion in laying hen diets.

## 6. Practical Implications

Overall, moderate inclusion of crude glycerol (CG) generally maintained or modestly improved productive performance without clear adverse effects on egg quality. However, the magnitude of these improvements was relatively small, suggesting that their practical significance under commercial production conditions may be limited. In contrast, some albumen-related traits appeared more sensitive at higher inclusion levels, indicating that responses may approach physiological or formulation limits as CG increases.

From a practical feeding standpoint, CG can serve as a partial energy substitute with minimal risk to egg quality when used at moderate inclusion levels. Nevertheless, its economic value is likely to depend on its relative cost compared with conventional energy sources such as maize or wheat, as well as its quality, consistency, and regional availability. Variability in glycerol purity and residual components may further influence its nutritional value and economic efficiency.

It is important to note that the primary studies included in this meta-analysis did not consistently report economic indicators such as feed cost or cost–benefit ratios, which limited the ability to perform a quantitative economic evaluation; however, biodiesel production is high, which makes CG production available at a lower price compared to conventional energy sources like corn. Therefore, the economic feasibility of CG inclusion should be assessed within specific production systems, accounting for ingredient pricing, diet formulation strategies, and production scale. Generally, the high between-study variability indicates that outcomes will depend on CG quality, diet formulation, bird genotype, and production stage, and further economic validation under commercial conditions is warranted before broad adoption.

## Figures and Tables

**Table 1:** Characteristics of studies used for meta-analysis.

No.	Study	Country	Strain	Inclusion Level (%)	Number of Birds	Crude Glycerol Composition		Studied Laying Age (wk)	Outcomes
						% Glycerol	% Methanol		
1	[11]	Poland	Bovans Brown	0, 2, 4, 6	72	-	-	28–53	1, 2
2	[17]	Turkey	Lohmann Brown	0, 2.5, 5, 7.5	180	90%	NS	39–55	1, 2, 3
3	[32]	Poland	Bovans Brown	0, 4	144	-	-	81–100	1, 2
4	[33]	Hungary	Tetra SL	0, 5, 7.5, 10	64	86.8%	0.04%	28–37	1, 2
5	[34]	Czech Republic	Hisex Brown hybrid	0, 2, 4	72	80%	-	20–44	1, 2
6	[12]	Brazil	Hy-Line W36	0, 1.5, 3, 4.5, 6, 7.5	240	-	-	35–51	1, 2, 3
7	[21]	Brazil	Hy-Line W36	0, 1.5, 3, 4.5, 6, 7.5	240	55.45%	0.05%	39–55	1, 2, 3
8	[35]	Spain	Lohmann Brown	0, 7	480	81%	-	23–51	1, 2
9	[18]	Turkey	Hy-Line W36	0, 0.725, 2, 4	60	90%	0.3%	44–56	1, 2, 3
10	[36]	Brazil	Hy-Line Brown	0, 2, 4, 6, 8, 10	252	76.5%	0.16%	90–102	1, 2
11	[19]	Turkey	Hy-Line W36	0, 0.8, 2.15, 5.02	60	-	-	44–56	1, 2, 3
12	[37]	Turkey	Backcob Brown	0, 3, 6, 9	320	84.2%	0.002%	20–40	1, 2
13	[14]	Brazil	Hy-Line W36	0, 7, 14	320	-	0.06%	39–57	1, 2

1, Production performance; 2, Egg quality; 3, Egg yolk fatty acid. -, not reported; NS, not specified in the original publication.

**Table 2:** Effects of dietary crude glycerol on production performance of laying hens, *n* = 13 studies.

Outcome ^1^	NC	MD	95% CI	*p*-Value	τ^2^	I^2^
EP	35	0.707	0.117 to 1.296	0.019	2.031	89.2
FI	35	0.475	−0.568 to 1.517	0.372	7.685	96.0
FCR	35	−0.002	−0.020 to 0.015	0.813	0.002	88.8

^1^ EP, Egg production %; FI, feed intake g/h/d; FCR, feed conversion ratio (kg feed/kg egg); NC, number of comparisons; MD, mean difference; CI, confidence interval; τ^2^, Cochran’s Q statistic; I^2^, inconsistency index.

**Table 3:** Effects of dietary crude glycerol on egg quality traits, *n* = 12 studies.

Outcome	NC	MD	95% CI	*p*-Value	τ^2^	I^2^
Egg weight (g)	38	−0.027	−0.355 to 0.301	0.872	0.646	92.6
Egg mass (g)	23	1.083	0.303 to 1.862	0.007	1.980	82.8
Albumen height (mm)	7	−0.137	−0.231 to −0.042	0.004	0.007	77.3
Haugh unit	25	−0.408	−0.879 to 0.063	0.089	1.000	91.4
Yolk colour (score)	8	0.012	−0.020 to 0.044	0.466	0.000	3.83
Egg shell thickness (mm)	17	0.002	−0.004 to 0.007	0.568	0.000	72.5
Egg shell breaking strength (N)	13	−0.175	−1.352 to 1.001	0.770	0.000	0
Egg shell (%)	16	−0.036	−0.081 to 0.010	0.123	0.004	52.6

NC, number of comparisons; MD, mean difference; CI, confidence interval; τ^2^, Cochran’s Q statistic; I^2^, inconsistency index.

**Table 4:** Effects of dietary crude glycerol on egg yolk fatty acid composition (% of total fatty acids), *n* = 5 studies.

Outcome	NC	MD	95% CI	*p*-Value	τ^2^	I^2^
Myristic (C14:0)	13	0.048	0.026 to 0.070	<0.001	0.000	86.7
Palmitic (C16:0)	19	−0.441	−0.581 to −0.300	<0.001	0.069	99.5
Stearic (C18:0)	19	−0.122	−0.237 to −0.007	0.037	0.032	96.4
Oleic (C18:1)	19	0.243	0.025 to 0.460	0.029	0.145	99.0
Linoleic (C18:2)	16	1.768	1.463 to 2.074	<0.001	0.322	98.7
Linolenic (C18:3)	16	−0.049	−0.108 to 0.011	0.108	0.008	98.4
Arachidic (C20:0)	13	−0.010	−0.025 to 0.004	0.160	0.000	94.0
Behenic (C22:0)	13	0.062	0.015 to 0.110	0.010	0.006	95.1
Docosahexaenoic (C22:6)	13	0.001	−0.002 to 0.004	0.540	0.000	0
Lignoceric (C24:0)	13	0.010	0.003 to 0.017	0.006	0.000	98.9
SFA	19	−0.346	−0.592 to −0.100	0.006	0.246	99.0
UFA	13	1.668	0.766 to 2.569	<0.001	2.613	98.6
MUFA	19	0.346	−0.035 to 0.726	0.075	0.548	99.4
PUFA	19	0.799	0.411 to 1.188	<0.001	0.489	99.7
PUFA/SFA	13	0.048	0.034 to 0.061	<0.001	0.001	96.3

NC, number of comparisons; MD, mean difference; CI, confidence interval; τ^2^, Cochran’s Q statistic; I^2^, inconsistency index.

**Table 5:** Subgroup analyses of the effect of covariates of CG on the performance of laying hens.

Outcome	Moderators	Subgroup	K	NC	MD	95% CI	*p*-Value
FCR	Inclusion levels	≤4%	13	15	−0.002	−0.033 to 0.028	0.878
		>4%	10	20	−0.000	−0.022 to 0.021	0.978
	Laying age	<57 wk	11	29	−0.003	−0.021 to 0.015	0.739
		>57 wk	2	6	0.044	−0.087 to 0.174	0.512
	Strain	Brown-egg	7	18	0.034	−0.003 to 0.071	0.071
		White-egg	6	17	−0.022	−0.038 to −0.006	0.006
EP	Inclusion levels	≤4%	13	15	0.930	−0.214 to 2.074	0.111
		>4%	10	20	0.517	−0.136 to 1.171	0.121
	Laying age	<57 wk	11	29	0.360	−0.238 to 0.958	0.238
		>57 wk	2	6	4.630	2.903 to 6.356	<0.001
	Strain	Brown-egg	7	18	1.694	1.090 to 2.298	<0.001
		White-egg	6	17	−0.072	−0.817 to 0.674	0.851
HU	Inclusion levels	≤4%	13	12	−0.621	−1.519 to 0.277	0.175
		>4%	10	13	−0.286	−0.811 to 0.240	0.286
	Laying age	<57 wk	11	24	−0.405	−0.880 to 0.069	0.094
		>57 wk	2	N/A	−0.700	−4.738 to 3.338	N/A
	Strain	Brown-egg	7	8	−1.005	−1.409 to −0.601	<0.001
		White-egg	6	17	−0.229	−0.828 to 0.370	0.454
EM	Inclusion levels	≤4%	13	10	1.273	0.253 to 2.293	0.014
		>4%	10	13	1.013	−0.037 to 2.062	0.059
	Laying age	<57 wk	11	17	0.668	−0.185 to 1.521	0.125
		>57 wk	2	6	2.681	1.280 to 4.082	<0.001
	Strain	Brown-egg	7	16	0.948	0.111 to 1.785	0.026
		White-egg	6	7	1.268	0.130 to 2.406	0.029
Linoleic (C18:2)	Inclusion level	≤4%	5	9	0.596	0.045 to 1.148	0.034
		>4%	4	10	1.296	−0.720 to 3.312	0.208
	Strain	Brown-egg	1	3	0.601	0.124 to 1.077	0.014
		White-egg	4	16	1.216	0.865 to 1.567	<0.001
PUFA	Inclusion level	≤4%	5	9	0.358	−0.265 to 0.981	0.260
		>4%	4	10	1.289	−0.257 to 2.836	0.102
	Strain	Brown-egg	1	16	0.737	0.202 to 1.272	0.007
		White-egg	4	3	0.794	0.366 to 1.221	<0.001

NC, number of comparisons; MD, mean difference; CI, confidence interval; FCR, feed conversion ratio (kg feed/kg egg); EP, egg production, %; HU, Haugh unit; EM, egg mass (g). For FCR, negative; K, number of studies; MD indicates improved feed efficiency. *p*-Values are for pooled effects within subgroups. N/A, not available due to insufficient number of studies.

## Data Availability

Data supporting this study are available from the corresponding author upon reasonable request.

## Supplementary Materials

The following supporting information can be downloaded at: https://www.mdpi.com/article/10.3390/ani16101486/s1, Data S1. PRISMA 2020 Checklist, Data S2. SYRCLE risk of bias Table. Ref. [55] is cited in Supplementary Materials.
